# Anatomy of a glacial meltwater discharge event in an Antarctic cove

**DOI:** 10.1098/rsta.2017.0163

**Published:** 2018-05-14

**Authors:** Michael P. Meredith, Ulrike Falk, Anna Valeria Bers, Andreas Mackensen, Irene R. Schloss, Eduardo Ruiz Barlett, Kerstin Jerosch, Adrián Silva Busso, Doris Abele

**Affiliations:** 1 British Antarctic Survey, High Cross, Madingley Road, Cambridge CB3 0ET, UK; 2 University of Bremen, Bremen, Germany; 3 Alfred Wegener Institute, Helmholtz Centre for Polar and Marine Research, Am alten Hafen 24/Am Handelshafen 12, 27570 Bremerhaven, Germany; 4 Instituto Antártico Argentino, Buenos Aires, Argentina; 5 Centro Austral de Investigaciones Científicas (CADIC, CONICET), Ushuaia, Argentina; 6 Universidad Nacional de Tierra del Fuego, Ushuaia, Argentina; 7 University of Buenos Aires, 1053 Buenos Aires, Argentina

**Keywords:** glacial discharge, Antarctica, geochemical tracers, stable isotopes

## Abstract

Glacial meltwater discharge from Antarctica is a key influence on the marine environment, impacting ocean circulation, sea level and productivity of the pelagic and benthic ecosystems. The responses elicited depend strongly on the characteristics of the meltwater releases, including timing, spatial structure and geochemical composition. Here we use isotopic tracers to reveal the time-varying pattern of meltwater during a discharge event from the Fourcade Glacier into Potter Cove, northern Antarctic Peninsula. The discharge is strongly dependent on local air temperature, and accumulates into an extremely thin, buoyant layer at the surface. This layer showed evidence of elevated turbidity, and responded rapidly to changes in atmospherically driven circulation to generate a strongly pulsed outflow from the cove to the broader ocean. These characteristics contrast with those further south along the Peninsula, where strong glacial frontal ablation is driven oceanographically by intrusions of warm deep waters from offshore. The Fourcade Glacier switched very recently to being land-terminating; if retreat rates elsewhere along the Peninsula remain high and glacier termini progress strongly landward, the structure and impact of the freshwater discharges are likely to increasingly resemble the patterns elucidated here.

This article is part of the theme issue ‘The marine system of the West Antarctic Peninsula: status and strategy for progress in a region of rapid change’.

## 1. Introduction

Since the middle of the last century, the West Antarctic Peninsula (WAP) has shown strong atmospheric warming, with marked variability and periods of cooling superposed [1]. Rates of warming up to the late 1990s were among the strongest globally, and were associated with rapid retreats of sea ice, surface ocean warming and a shortening of the sea ice season [2–4]. While rates of change have subsequently been markedly lower, the WAP remains an area of profound interest concerning climatic change and its impacts on the marine environment [5]. Concurrent with the atmospheric and sea ice changes has been a retreat of the majority of marine-terminating glaciers along the WAP, and a recent acceleration in their retreat rates [6]. This was initially presumed to be causally linked to the atmospheric warming and southward progression of isotherms, but it was recently shown that strongest retreats have occurred predominantly in the central/southern WAP region where intrusions of warm, deep water from offshore can penetrate across the shelf and undercut the marine termini of the glaciers [7]. Further north on the WAP shelf, where deep waters are significantly cooler, there is not the same consistent pattern of retreat; nonetheless, these glaciers remain of significant influence not least because of the physical and geochemical influence they exert on the ocean [8,9].

**Figure 1.**
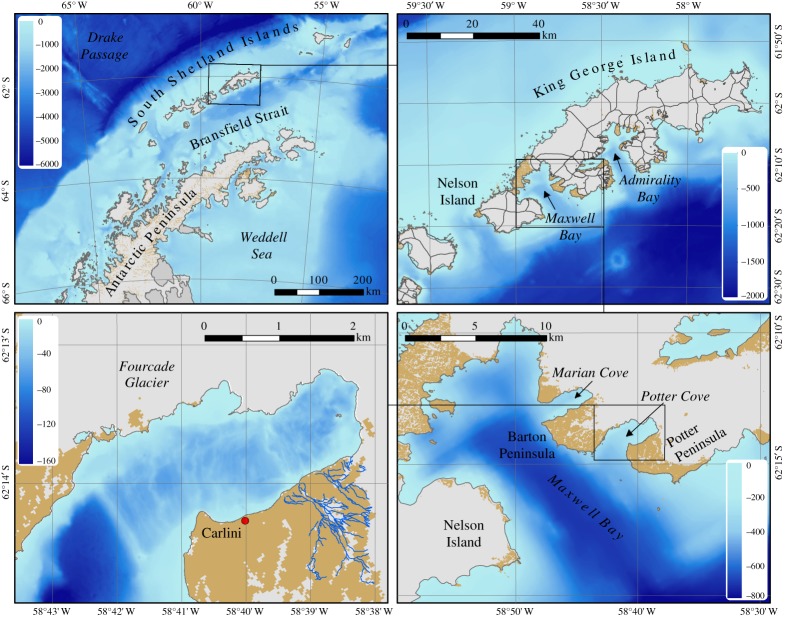
Maps showing location and bathymetry of Potter Cove, Maxwell Bay and the environs of King George Island at the northern end of the Antarctic Peninsula. Panels focus on progressively smaller scales, clockwise from top left. Arrows indicate bays and coves as labelled. Areas marked in brown denote land not covered by ice; this is not necessarily bedrock but includes surfaces with soil formation, sand and rubble. Blue lines in lower left panel denote the paths of meltwater streams.

At the very northern tip of the WAP, the area of Bransfield Strait and the South Shetland Islands (figure 1) is influenced atmospherically by the westerly winds that overlie the Southern Ocean, and which have been intensifying in recent decades as a consequence of more frequent positive phases of the summer Southern Annular Mode (SAM) [10]. These winds drive warm and moist air toward and across the northern WAP, where they cause surface thinning and disintegration of the coastal ice shelves with resulting acceleration of the coastal marine and tidewater glaciers [11,12]. Once the buttressing ice shelf has been removed, the speed of glacial mass loss becomes a function of surface and basal melting rates of the local ice sheet margins. Atmospheric warming and precipitation cause surface thinning and meltwater infiltrations into the icecap which add to basal meltwater formation and accelerate glacial ice flow [13]. Glaciers on the smaller island icecaps, such as on King George Island/25 de Mayo (KGI; figure 1), are currently retreating at unprecedented speed [14,15]. The immediate consequences of glacier and ice sheet loss at the northern WAP are diverse, and include a modification of ocean stratification and circulation, and changes to oceanic light levels and primary productivity [16]. Furthermore, the Peninsula glaciers north of 70° S have the potential to raise sea level by 69 ± 5 mm [17].

The occurrence of extended meltwater plumes from northern WAP glaciers can transport large quantities of lithogenic particles derived from subglacial erosion and ice-free surface outwash into near-coastal and shelf areas. This can represent an enhancement of micronutrient supply to the near-coastal and high nutrient/low chlorophyll regions of the Southern Ocean [18], especially the bioavailable forms of iron [8]. Increased appreciation of the importance of these phenomena has led to renewed interest in determining the key processes and impacts that occur in the coves and fjords that connect the northern WAP glaciers to the broader ocean, and which are currently insufficiently understood and often inadequately represented in models.

A long-term interdisciplinary research programme at KGI has focused on the multiple drivers and interactive effects of the melting Fourcade Glacier that drains into its coastal fjord, Potter Cove, and from there transiting Maxwell Bay into Bransfield Strait (figure 1). The Fourcade Glacier is currently retreating at around 40 ma^−1^, becoming land-terminating in 2016. Potter Cove circulation is generally cyclonic, and is influenced by the circulation of the adjacent Maxwell Bay that reaches down to 500 m depth [19,20]. Strong katabatic winds can mix the water column down to the seabed, which can resuspend the soft sediments present in the inner cove. Horizontal circulation is noted to be significantly wind-forced, with indications of upwelling of deep waters in the cove's inner part under northeasterly winds, while tides may be significant in modulating circulation during periods of comparatively weak wind forcing (less than 4 ms^−1^) [20].

Approximately 20 700 m^3^ of glacier ice is discharged into Potter Cove per day during the melt season, with a broadly comparable amount of meltwater drainage [21]. A turbid freshwater layer approximately 5 m thick forms at the surface of the cove during the melt season. This layer is generated by cascading glacial surface melt and subglacial meltwater entering the inner cove mainly in its northeastern section where the receding ice was, at the time of sampling, still directly bordering and adjacent to the water (marked white in figure 1, with brown areas indicating ice-free coastal areas). Floating ice blocks and brash ice, melting within the plume or onshore, enhance the freshness of this surface layer. Mixed discharge of surface and subterraneal meltwater streams enter Potter Cove at its southern coastline [9]. Part of the subterraneal discharge occurs at shallow depths and is a source of bioavailable aqueous Fe(oxyhydr)oxides or reactive ferrihydrite nanoparticles to the shallow mixed layer [22,23]. Subglacial iron-bearing meltwaters and iron leaching from suboxic coastal and shelf sediments at the South Shetland Islands is an important source for natural fertilization of the Scotia Sea through advective transport [24,25].

Locally, the turbid meltwaters tend to have an adverse effect on productivity by restricting light penetration into the seawater, thus constraining benthic and pelagic primary production in the water column. Potter Cove has thus traditionally been regarded as a low productivity ecosystem with short bloom events, sometimes lasting only a few days to weeks, with maximum Chl *a* values around 4 mg m^−3^ [26]. Strong bloom events are seen only after very cold winters (e.g. El Niño years) when late disintegration of coastal sea ice stabilizes surface stratification before the onset of glacial discharge flow [16]. Only few macroalgal species colonizing clear water areas in the outer cove are found on the newly ice-free hard substrates in glacial vicinity; these species need to be adapted to low light conditions to manage with only a short growth period in early spring [27]. Species adapted to low light are also typical representatives of the pelagic microbial pro- and eukaryote communities (see [28] for review).

There is thus a strong need to understand better the physical drivers and ecological impacts of glacial discharge into coves and fjords at localities such as the northern WAP. Accordingly, we conducted a short, dedicated sampling programme to resolve the three-dimensional structure of the glacial meltwater plume in Potter Cove and the opening into Maxwell Bay with unprecedented spatial and temporal resolution. Serendipitously, this programme captured the initiation, evolution and fate of a pronounced meltwater discharge and outflow event, and hence offers unique insight into the drivers and controls on the glacially derived freshwater.

## 2. Material and methods

### (a) Sample and water column data collection

**Figure 2.**
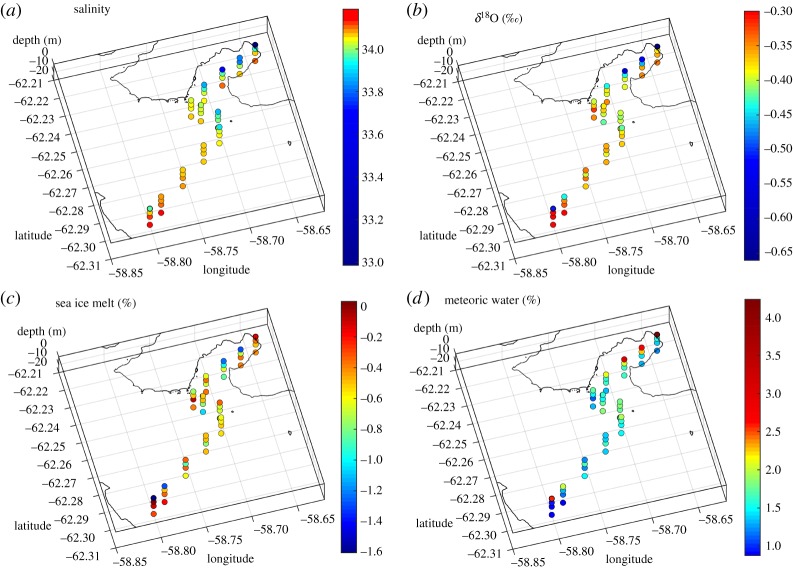
Perspective maps of (*a*) salinity, (*b*) *δ*^18^O, (*c*) sea ice melt, (*d*) meteoric water across Maxwell Bay and Potter Cove. Data are from samples collected on 16 February 2013.

**Figure 3.**
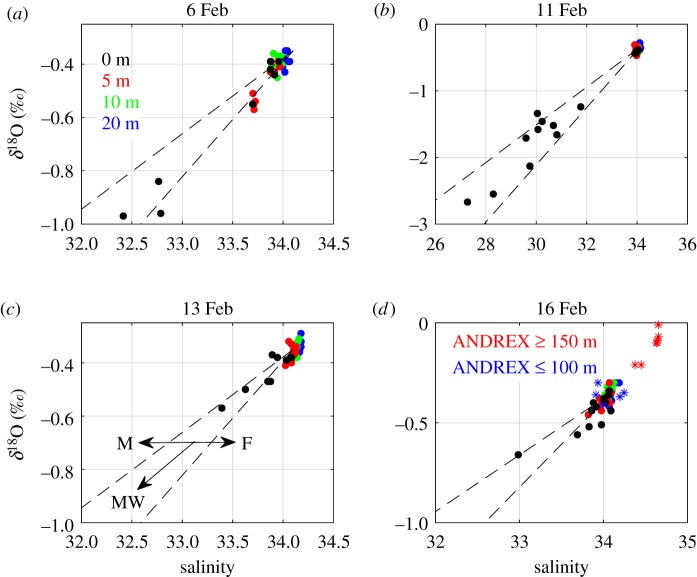
Salinity versus *δ*^18^O for samples collected across the full period of fieldwork presented here. Note the expanded scale for 11 February (figure 3*b*) to incorporate the very fresh, isotopically light waters present then, and for 16 February (figure 3*d*) to illustrate the conditions on the Peninsula shelf outside Maxwell Bay, as reflected in the 2010 ANDREX data (red and blue asterisks). In figure 3*c*, M denotes sea ice melting, F denotes sea ice freezing and MW denotes addition of meteoric water.

Water samples for oxygen isotope analysis were collected at four depths (0, 5, 10 and 20 m) along transects across Potter Cove and Maxwell Bay, using 4.7 l Niskin bottles. Concurrent water column profiling was conducted using a Sea-Bird SBE 19 conductivity–temperature–depth (CTD) instrument, with an auxiliary sensor of turbidity ECO NTU. Salinity profiles were derived from the CTD data, and values extracted from the levels corresponding to the depths of the water sampling. Turbidity data were here averaged over the upper 3 m of the water column for analysis. Sampling events were conducted on each of 6, 11, 13 and 16 February 2013. The spatial pattern of sampling and data coverage is indicated by the distributions of salinity and isotopes shown in figures 2 and 3.

### (b) Oxygen isotope measurement

From each Niskin event, samples of 100 ml water were drawn into glass vials, sealed with wax and stored at 4°C temperature prior to analysis. In the laboratory, 7 ml of water was equilibrated in 13 ml headspace with CO_2_ gas using a Finnigan equilibration device. Oxygen isotope equilibrium in the CO_2_–H_2_O system was attained by shaking for 430 min at 20°C. The equilibrated gas was purified and transferred to a Finnigan Delta-S gas mass spectrometer. Sample preparation and isotope measurements were calibrated against Vienna Standard Mean Ocean Water (VSMOW) and Standard Light Antarctic Precipitation (SLAP) standard waters. At least two replicates (including preparation and measurement) were run for each oxygen isotope determination. Results are reported in δ-notation (*δ*^18^O) versus VSMOW with a mean precision better than ±0.03‰. Fuller details are provided in [29].

### (c) Identification and quantification of meteoric water

The benefit of *δ*^18^O, when measured in addition to salinity, is that it permits identification of meteoric-derived freshwater (i.e. that derived from glacial sources and/or precipitation) separately from sea ice melt. This is because both meteoric water injection to the ocean and sea ice production/melt have significant impacts on ocean salinity, whereas only the former has a significant impact on ocean *δ*^18^O. Fuller background on *δ*^18^O as a freshwater tracer, and its application at the WAP, is available in [30].

For each *δ*^18^O value, corresponding salinity was extracted from the CTD data and the pairs of data were used in a mass balance that presumes the composition of each sample to be a simple mixture of three components, namely sea ice melt (sim), meteoric water (met) and ocean water (ow):-
2.1
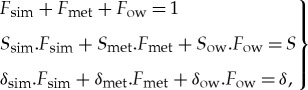

where *S*_sim_, *S*_met_, *S*_ow_ are the endmember salinities of the source components; *δ*_sim_, *δ*_met_, *δ*_ow_ are the corresponding *δ*^18^O values; and *S*, *δ* are the measured salinity and *δ*^18^O of each sample. This system of equations is solved for *F*_sim_, *F*_met_, *F*_ow_, which are the respective fractions of the three components in each sample collected.

Application of this method requires the endmember values of the unmixed source waters to be established. Here we use values of *S*_ow_ = 34.40, *δ*_ow_ = −0.2‰, following data presented in [31] for properties in Maxwell Bay. *S*_met_ is taken to be 0.0, and *δ*_met_ is set to −11‰, following [32,33]. This value varies from others used in isotopic studies further south along the WAP [30,34], consistent with the spatially varying *δ*^18^O structure of the Peninsula glaciers. We also use *S*_sim_ = 5, which is a reasonable approximate value for this region, and *δ*_sim_ is set as +1.6‰ to take into account fractionation processes upon sea ice formation. Typical uncertainties using this method are close to or less than ±1% in the freshwater fractions quantified, and derive mainly from uncertainty in the meteoric water endmember choice [30].

### (d) Meteorological data

An Automatic Weather Station (AWS, Campbell Scientific, Logan, USA) was installed at 62°14'09.8′′ S, 58°36'48.7′′ W (230 m above sea level) on the Fourcade Glacier, and operated from November 2010 until March 2017 [35]. The AWS was designed to estimate all components of the surface energy balance equation and equipped with wind anemometers and vanes (Alpine Wind Monitor) at two heights, air temperature and relative humidity sensors (HMP155A), and five depths of snow and ice temperature measurements (107 Thermistor Probes) to derive sensible, latent and ground (ice) heat fluxes. The AWS included a four-component radiation sensor for up- and downwelling longwave and shortwave radiation fluxes (NR01), in addition to a SR50A Sonic Ranging Sensor installed at an initial height of 2 m to measure surface elevation changes. Levelling and adjustment of sensors were carried out at the start and end of each summer field campaign. In particular, the whole station needed to be lowered about 2–3 m at the end of the ablation season each year. Measurement rate was set to every 5 s with an averaging interval of 10 min.

### (e) Glacial discharge modelling

The meteorological data were aggregated to hourly values and gap-filled in order to force a glaciological melt model developed by [36] and adapted for the South Shetland Islands by [15] to assess glacial discharge from Fourcade Glacier into Potter Cove. The glacier melt model calculates the energy available for melt as the residual of the surface energy balance equation:
2.2
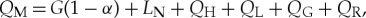

where *L*_N_ is the longwave net balance, *G* the global radiation. The term (1 − *α*) signifies the solar incidental radiation minus the part (*α* = albedo) reflected at the glacier surface. *Q*_H_ and *Q*_L_ are sensible and latent heat, respectively, and *Q*_G_ is the ground heat flux. *Q*_R_ is the sensible heat supplied by rain during phase transition from liquid to solid.

The energy balance equation is solved for each time step and each point on a spatial grid of 10 m by 10 m, taking into account the differing exposure of the glacier surface, zonal distinction of ablation and accumulation patterns [21] and surface properties and lapse rates of air temperature, precipitation and wind speed. The albedo *α* is defined by the surface’ ablation and accumulation pattern and is configured to reflect the alteration of the snow pack with time. The sum of computed melt and rain water is integrated over the glacier catchment area, and defines the glacial discharge. Output of the model is glacier discharge calculated on an hourly basis. Although calibration and validation processing show a good agreement between model and observations, the processes of turbulence-driven snow deposition, refreezing and wind drift are not taken into account by the model. The model thus overestimates glacial discharge, predominantly at the end of the melt season, i.e. March to May. Turbulence-driven snow deposition prevails in the vicinity of obstacles, e.g. moraines and steep topography, and is confined locally. This process is not incorporated in the model physics. It adds to the uncertainties in model output. During the here considered time period, uncertainty in simulated glacial discharge can be assumed to be less than 10%.

## 3. Results

### (a) Isotopic structure of Potter Cove and Maxwell Bay waters

The most spatially expansive sampling for oxygen isotopes and oceanographic parameters was conducted on 16 February 2013, when a section was completed along the length of Potter Cove and extending across Maxwell Bay to reach close to Nelson Island (figure 2). The freshest waters encountered were at the surface near the head of Potter Cove (salinity around 33.0); waters were progressively more saline with depth and towards the centre and western flank of Maxwell Bay, but with some slightly fresher waters (salinity around 33.9) at the surface close to Nelson Island (figure 2*a*). This general pattern is mimicked by that of *δ*^18^O, which had values around −0.5 to −0.6‰ at the surface near the head of Potter Cove, with higher values (−0.3 to 0.4‰) at greater depths and extending toward the centre of Maxwell Bay (figure 2*b*). Adjacent to Nelson Island there is some evidence of isotopically lighter waters at the surface (−0.45 to −0.6‰).

Applying equation (2.1) to these data reveals the spatial fields of sea ice melt and meteoric water (figure 2*c,d* respectively); this demonstrates that meteoric water is strongly responsible for the low salinities observed at the surface of Potter Cove, with 3–5% of the waters sampled being of meteoric origin at this time. Sea ice melt values are generally lower and more scattered, with slight negative values denoting small net sea ice production from the waters sampled relative to the endmembers chosen (figure 2*c*).

These freshwater contributions are also evident when the data are viewed in salinity-δ^18^O space (figure 3*d*), which shows a cluster of points from the subsurface layers (red, green and blue dots) near S = 34, *δ*^18^O = −0.35‰, but with the surface layer (black dots) showing an extension along the meteoric water mixing line, and with some points offset from this line slightly toward higher salinity. (The arrows in figure 3*c* indicate the impact that freshwater inputs will have on the locus of data points, with sea ice melt (freeze) moving the locus horizontally to the left (right), while meteoric water injection would move the locus diagonally downward to the left.)

Also shown in figure 3*d* are data points from a research cruise that was conducted in 2010 (ANDREX; [37]), during which *δ*^18^O was measured across Bransfield Strait and the northern Antarctic Peninsula shelf. The deeper ANDREX data (red asterisks; depths greater than 150 m) show data that are more saline and isotopically heavier than were measured in the shallower layers across Maxwell Bay; however, the shallower ANDREX data (blue asterisks; depths less than 100 m) lie on the same cluster near S = 34, *δ*^18^O = −0.35‰. This indicates that the processes which mediate the general exchange of waters between Maxwell Bay and the adjacent shelf do not result in major water mass modification, and that water discharged from peripheral coves into Maxwell Bay can penetrate outwards to the broader shelf relatively uninhibited.

### (b) Changes in the composition of Potter Cove waters

Figure 3*a–d* shows the full sequence of data collected within Potter Cove during each of February 6, 11, 13 and 16, respectively. The general pattern of subsurface waters is consistent across each day of sampling, clustering on a comparatively saline, isotopically heavy point. By contrast, while data in the surface layers all extend toward fresher, isotopically lighter waters, the extent to which they do this varies dramatically. In particular, 11 February shows remarkably fresh, isotopically light surface properties (S < 28.0; *δ*^18^O < −2.5‰) compared with preceding or subsequent days. Full-resolution CTD profile data (not shown) reveal this to be an extremely thin (less than 1 m) layer.

**Figure 4.**
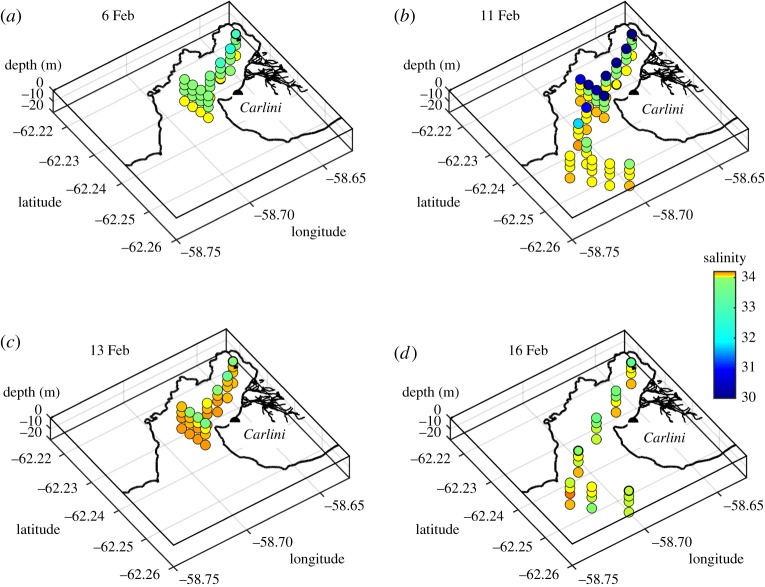
Salinity in Potter Cove for (*a*) 6 February 2013, (*b*) 11 February 2013, (*c*) 13 February 2013 and (*d*) 16 February 2013. Note, in particular, the very fresh surface layer present on 11 February.

**Figure 5.**
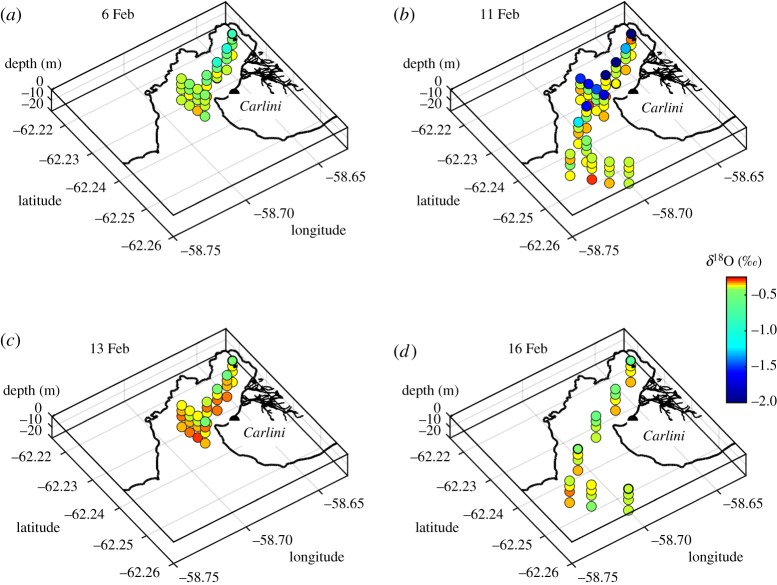
As per figure 4, but for *δ*^18^O. Note, in particular, the very isotopically light surface layer present on 11 February.

The spatial freshwater structure is evident in figure 4, the most striking feature of which is the very low salinity layer that occupied the surface of most of Potter Cove on 11 February (figure 4*b*). While the previous sampling on 6 February (figure 4*a*) was not as spatially extensive as that on 11 February, there was no evidence for this layer in the inner part of Potter Cove at that time. Just two days after its detection (13 February; figure 4*c*), the layer had disappeared, and there is evidence that salinities within the inner part of Potter Cove were elevated at depth relative to those observed prior to the layer's creation (cf. 6 February). More expansive sampling on 16 February (figure 4*d*) revealed no evidence of the layer, and conditions were generally similar to those at the start of the data sequence (February 6^th^). The *δ*^18^O data from the sequence of samplings (figure 5) show patterns that strongly resemble the salinity data, consistent with the inferred dominance of meteoric water inputs in determining the freshwater structure of our data.

**Figure 6.**
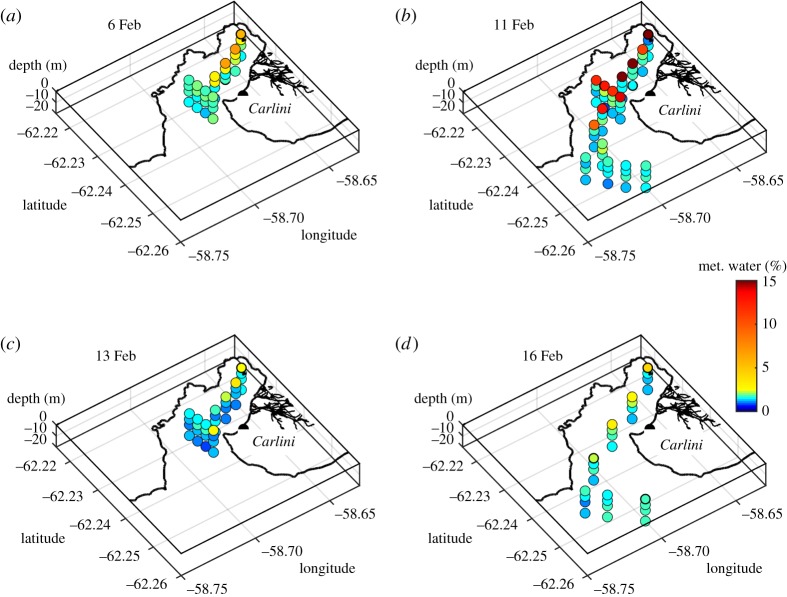
As per figure 4, but for meteoric water percentage calculated according to equation (2.1). Note, in particular, the very high levels of meteoric water present on 11 February.

Using equation (2.1), we quantify the meteoric water prevalence across Potter Cove as a function of time (figure 6). This shows moderate values at the surface on 6 February (figure 6*a*; maximum around 5%), rising sharply to approximately 15–20% on 11 February (figure 6*b*) before dropping dramatically thereafter. Sea ice melt prevalences (not shown) are very much smaller throughout the full sequence of measurements, with values in the range −2 to + 1%; the oxygen isotopes thus confirm the negligible impact of sea ice in contributing to the freshwater event.

**Figure 7.**
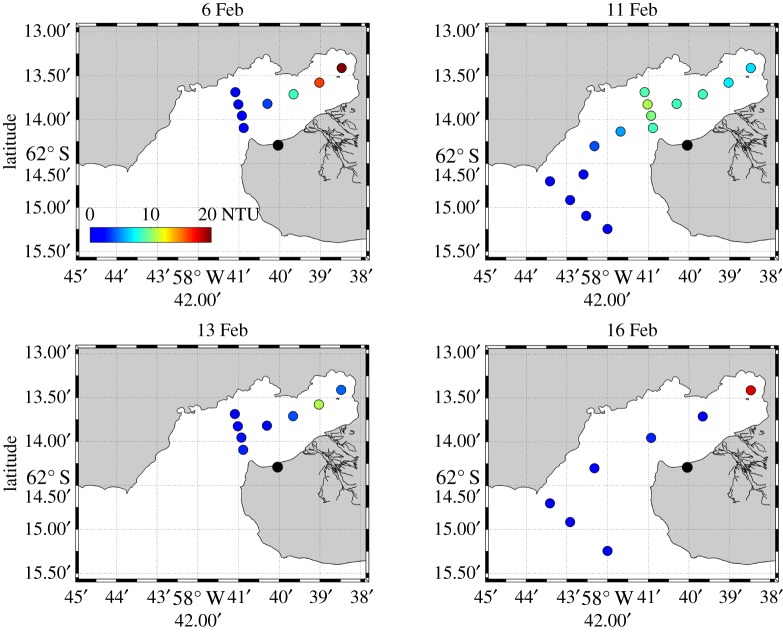
Turbidity (NTU) averaged over the upper 3 m of Potter Cove during the sequence of measurements. Note that the spatial extent of elevated turbidity on 11 February (figure 7*b*) coincides with the strong prevalence of meteoric water at that time (figure 6*b*).

Turbidity also showed marked changes during our sequence of data (figure 7). Initial values (6 February; figure 7*a*) were moderately low except at the very head of Potter Cove. When the strong freshwater layer occupied the surface of Potter Cove (11 February), the turbidity values were generally higher (values around 10 NTU), with the exception of at the head of the cove (figure 7*b*). Subsequent to the loss of the strong freshwater layer (13 and 16 February; figure 7*c,d*), the turbidity values in the cove declined, reaching a minimum on 16 February except in the immediate proximity of the glacier head where subglacial melt leaks into the cove.

### (c) Meteorological and glaciological forcings

**Figure 8.**
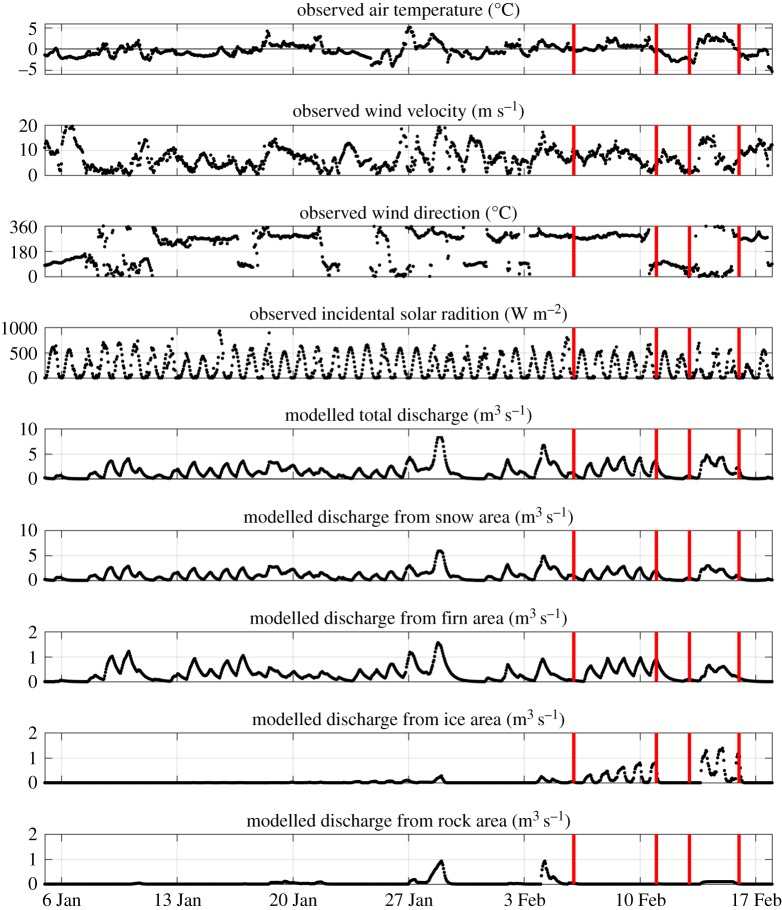
Meteorological and glacial discharge series for the period of January–February 2013. Discharge data are calculated as described in the text. Vertical red bars denote the timing of collection of isotope samples and oceanographic data.

Figure 8 shows the key meteorological data from the period under study, and the modelled meltwater discharge from the different components of the Potter Cove glacial system. Prior to our initial isotope sampling on 6 February, there had been a general rise in air temperature of 1–2°C since the start of January 2013, though with significant variability superposed. Associated with this, there had been a general increase in meltwater discharge, with discharge from the snow area dominating.

The changes in air temperature were strongly coupled with variability in wind direction. In general, winds from the west are associated with advection of warm and moist air masses from mid latitudes, whereas winds from the east are associated with katabatic wind systems and the influence of the Antarctic high pressure cell. Between 6 and 11 February, winds were consistently from the west, air temperature remained above the freezing point, and the rate of total glacial discharge remained significant. Between 11 and 13 February, winds were predominantly from the east, the air temperature dropped significantly and there was an associated marked decline in glacial discharge. After 13 February, the wind switched direction again, and became more variable.

The discharge calculated for the different source areas reveals the processes responsible for driving the discharge (figure 8). The firn area is in the glacier's accumulation zone, with air temperatures that are mostly below zero, while the snow area encompasses all surface areas with snow from the preceding winter. The main driver is the air temperature, and, to a lesser degree, the radiation flux densities due to high albedos of fresh snow and firn (approximately 0.75–0.9). The discharge in the ice area is driven to a large extent by radiation fluxes due to its significantly lower albedo (as low as less than 0.1), whereas the rock area shows the response to precipitation events.

## 4. Discussion and conclusion

Glacial discharge is known to exert a strong influence on marine physical, biogeochemical and ecological systems. A key factor that determines the nature of impact that glacial discharge will have is the depth at which it enters the ocean. Meltwater injected at depth may rise as a buoyant plume, but if it entrains oceanic water as it ascends it may reach a level of neutral buoyancy before it reaches the surface; at this point, the (bio)geochemical tracers contained therein may be restricted to lie below the euphotic zone. Conversely, meltwater discharged to the upper layers will likely reinforce stability and strengthen stratification, and tracers and particles contained therein could be retained in the near-surface layers more effectively; however, strong mixing and lateral advection/dispersion will affect the fate and downstream impact of these meltwaters.

We have seen a marked example of the latter case, where a layer of glacially derived meltwater flooded the surface of a northern Antarctic Peninsula cove, creating an extremely thin, buoyant, freshwater layer. Serendipitously, our sampling enabled the determination of the spatial structure of this layer across the cove and beyond, and, combined with the local meteorology and glaciology data, it also allowed the genesis, evolution and demise of the layer within the cove to be characterized.

Of significant note is the strongly episodic nature of the freshwater layer. This was caused by complex interactions of meteorological forcings. The discharge to the ocean is strongly dependent on atmospheric temperature, which fluctuates on characteristic weather timescales, as well as seasonal and longer periods. The build-up of the freshwater layer within the cove was initially promoted by retentive winds, but reversal of the wind direction then flushed the layer rapidly out of the cove into Maxwell Bay, and ultimately beyond.

Using conservative freshwater tracers allows us to quantify some key aspects of the glacial discharge. We find that average meteoric water percentages at the surface were of order 20% during the sampling when the freshwater layer was present, so (very approximately) the pure meltwater released had mixed fivefold in the period over which it was released. This has implications for the concentration of other tracers within that layer, such as micronutrients, which would also have been subject to the same level of mixing.

During the 5 days between isotope samplings over which the freshwater layer built up (6–11 February), the average discharge into Potter Cove was around 2.5 m^3^ s^−1^. In the absence of any wind-forced export or mixing, this discharge over such an interval would create a layer approximately 15 cm thick at the ocean surface. This layer would be fivefold thicker if the water therein were mixed to 20% of its initial pure freshwater state, as suggested by the isotopic mass balance calculation, or approximately 75 cm thick. It should be noted that discharge was non-zero for a couple of days prior to our initial isotope sampling on 6 February (figure 8), and the values derived here are acknowledged to be very coarse. Despite this, overall the quantitative nature of the freshwater layer (including its thickness) is seen to be consistent with quantifications of the freshwater injected to the ocean.

The horizontal circulation changes alluded to above are only one aspect of the time-varying Potter Cove system. In addition, there is evidence that the vertical (overturning) circulation also responded to the changes in forcing identified herein. In particular, figure 5 shows that concurrent with the export of isotopically light water from the surface of the cove during 11–13 February, there was an increase in *δ*^18^O values in the subsurface layers. This water is also more saline, and must have been drawn into the cove from further offshore toward Maxwell Bay, because there was no deep water with comparable properties resident in Potter Cove up to that time. Following the end of the export event (16 February; figure 5*d*), there was an apparent relaxation back toward initial conditions at all depths, presumably due to slumping of internal ocean layers as the export-favourable surface wind stress reduced.

The loss of the surface fresh, isotopically light layer between 11 and 13 February is ascribed here to horizontal export, with minimal impact from vertical mixing. This is excluded on the grounds that the winds were actually no stronger during the period that the freshwater was exported than they were before that period (only the direction had changed), and the creation of the layer had not been impeded. Further, if the freshwater was being redistributed in the vertical as opposed to the horizontal, there would be no significant change in freshwater column inventories derived from the salinity profiles, which is not the case.

The depth over which the isotopic changes with opposing signs occur is important: the outward export of fresh, isotopically light water is evidenced in the surface samples, but all samples below (5 m and deeper) show evidence of import of more saline, isotopically heavier waters to the cove. This identifies that while changing wind forcing has a significant impact on the speed and direction of the surface layer circulation in the cove, it also influences the deeper circulation in an opposing sense, with the ‘hinge point’ for the accelerated overturning sitting above or close to 5 m depth.

It is possible to estimate the acceleration of the overturning circulation in the cove during the period of export-favourable winds, albeit very approximately. The volume of the inner cove is around 140 × 10^6^ m^3^; if this whole part of the cove were replenished over a 2 day period (11–13 February), this would require an overturning rate of 1600 m^3^ s^−1^ (or 1.6 mSv). It should be noted that this back-of-the-envelope calculation only represents the *minimum* overturning necessary to replenish the waters of the inner cove; the actual rate that occurred could have been significantly higher. Further, if one assumes a sill depth separating the inner cove of around 20 m, with a 2 km cove width at this point, one has an effective ‘flux gate’ of 40 000 m^2^ through which the exchanged waters must pass. Using this, a figure for mean speed of throughflow of around 4 cm s^−1^ can be derived which would be needed to support the overturning rate derived above. This can be compared with the efflux speed inferred for the period between 11 and 13 February, during which the whole inner cove was cleared of isotopically light water. If the export distance were approximately 3 km, a minimum mean export speed of around 2 cm s^−1^ would be needed to flush the freshwater from the cove. While both these calculations are necessarily very coarse, the approximate agreement lends credence to these results being representative ballpark figures for overturning and export rates.

Previous studies have used oxygen isotope tracers to elucidate the freshwater system, including at King George Island (e.g. [31]); often such studies use single sampling events to characterize an area. Further south on the WAP, a long-term programme involves quasi-weekly sampling for oxygen isotopes, but normally only at a single site, and (until recently) with the bulk of the sampling conducted in the subsurface layer (15 m; [30]). The strong episodic nature of the freshwater system noted in our more extensive sampling highlights that such sampling protocols may miss key elements of the spatially- and temporally varying system if applied without knowledge of the scales that are required to be resolved. This is important if dynamical drivers and their responses are to be correctly identified, and if their representation in models is to be determined as robust.

We have observed that the freshwater layer that capped Potter Cove on 11 February had generally elevated levels of turbidity, albeit with values not as extreme as individual points found close to the glacier on 6 or 16 February. It is presumed that this indicates some level of suspended particulate material being injected to the cove, though the levels observed suggest that the concentrations in the freshwater released may not have been very high. The fate of these particles when the freshwater layer is exported from the cove is not well determined, but will be some combination of lateral export and sinking to the seabed. Previous investigations using sediment traps in this locality found that between 15% and 50% of suspended particulate material was exported horizontally [9]. In both cases of high and low sediment export rates, however, there are significant potential consequences, especially if this general pattern of export is recurrent over multiple fjordic systems around the fringes of Antarctica. Strong injection of sediment to the ocean associated with glacial discharge has been associated with negative consequences for zooplankton, including mass mortality events for Antarctic krill (*Euphausia superba*); this was deduced using data collected within Potter Cove [38], where krill populations have been notably absent in recent years. Further, the benthic ecosystem has also been demonstrated to be strongly impacted by sediment accumulation [39,40]. The episodic discharge and flushing we have witnessed represents a layer of complexity in the delivery and export of this sediment, affecting the timescale of its retention within the cove, and its ultimate fate.

A key feature of our findings is the strong meteorological control of glacial meltwater discharge and efflux to the general shelf, as opposed to stronger oceanographic control further south. In particular, the delivery of the meltwater to the ocean appears heavily dependent on atmospheric temperature, and its fate in the ocean depends markedly on the directionality of the winds. Atmospheric warming on the WAP has recently undergone a period of hiatus associated with natural variability, but if the strong warming witnessed during the second part of the twentieth century resumes, one could potentially expect further accelerations of freshwater injection from systems comparable to Potter Cove. If the majority of the glaciers further south along the WAP continue to retreat landward, it is likely that such systems will progressively resemble more closely the one observed here.

The directionality of the winds has been identified as a key control in the fate of the freshwater. While this is clearly dependent on the orientation of the cove studied, it is very possible that other coves will have similar dependencies, albeit potentially to different components of the vector winds. Like atmospheric temperature, winds at the Peninsula are known to be sensitive to large-scale modes of climatic variability, including the El Niño-Southern Oscillation phenomenon and the SAM [41]. Each of these has long-period variability, including a decadal trend in the SAM that is known to be at least partly driven by greenhouse gas emissions and (in particular) ozone depletion. This raises the likelihood of long-period (decadal) changes in both the discharge and fate of freshwater released into Antarctic coves including an element driven by anthropogenic forcings.

## Data accessibility

Bathymetry data are available at https://doi.pangaea.de/10.1594/PANGAEA.853593. Oceanographic (CTD) data are available at https://doi.org/10.1594/PANGAEA.869413.

Isotope data are available at https://doi.pangaea.de/10.1594/PANGAEA.884851. Glaciological datasets are available at https://doi.org/10.1594/PANGAEA.874599. Meteorological datasets are available at http://dx.doi.org/10.1594/PANGAEA.848704.
